# Supplementary material for: Successful treatment of recurrent visceral leishmaniasis relapse in an immunocompetent adult female with functional hypopituitarism in Bangladesh
Source: PLoS Negl Trop Dis. 2024 Apr 26;18(4):e0012134. doi: 10.1371/journal.pntd.0012134 (PMC11051618; doi:10.1371/journal.pntd.0012134)
Supplement: Attachment — Submitted filename: PLOS review.docx [file pntd.0012134.s001.docx]

I want to congratulate Dr. Tabiha and her team for their success in treating such a difficult case. Bangladesh recently accomplished the target of achieving VL elimination. This relapse VL case might be the source for future cases. Treating VL relapse cases, especially those refractory cases, is a great challenge. How the authors treat this case is wonderful. I think this regimen in the future will be an effective regimen for other refractory cases. The manuscript is well written and well organized. I again thank all the authors for their efforts.

I think this paper will guide clinicians in treating such cases in the future. I have some minor queries.

1. Has she established a natural menstruation cycle?
2. Is there any risk of developing drug resistance (LAMB or miltefosine)?
