# Supplementary material for: Successful treatment of recurrent visceral leishmaniasis relapse in an immunocompetent adult female with functional hypopituitarism in Bangladesh
Source: PLoS Negl Trop Dis. 2024 Apr 26;18(4):e0012134. doi: 10.1371/journal.pntd.0012134 (PMC11051618; doi:10.1371/journal.pntd.0012134)
Supplement: Attachment — Submitted filename: Reply to Reviewers_PLOS NTD_VL.docx [file pntd.0012134.s002.docx]

**Reply to Reviewers’ Comment**

**Reviewer #3: ‘Result is clear, although improvement in table format could be addressed.”**

- Thank you very much for your valuable input. I have split the table into two- one for general investigations, another one for endocrinological profile of the patients for better visualization. I hope this change makes it more palatable. Please see line 92.

**Reviewer #2: “Although the discussion of cases of therapeutic failure in leishmaniasis is of great interest to public health, the case presented in this manuscript failed to adequately discuss the impacts of alternative therapeutic regimens.”**

- Thank you very much for your valuable comment and suggestion. In recurrent VL relapse cases, extended or split dose Liposomal amphotericin B might not be enough to achieve complete cure, which increases the risk of future relapses. Multiple relapses eventually cause physical as well as economic burden to the patients, as this is a disease of the poor. So, in immunocompromised patients, secondary prophylaxis may be added to ensure complete cure. Since after treating our patient with loading dose followed by secondary prophylaxis, she developed menarche and her hormone profile (Thyroid status) also improved, this indicates towards the effective treatment of VL by secondary prophylaxis may aid in achieving complete cure. I have added these lines to the manuscript. Please see lines 123-125.

**Question 1: (Reviewer not identified) “Has she established a regular menstrual cycle?”**

- Thank you for your important query on this matter. Yes, she has developed regular menstrual cycle after 18 months of hormone replacement therapy with conjugated estrogen and progesterone. I have added these lines to the manuscript too. Please see the lines 100-102.

**Question 2: (Reviewer not identified) Is there any risk of developing drug resistance (LAMB or miltefosine)?**

- Thank you for your important query. Liposomal amphotericin B has a broad spectrum of activity, long half-life with a very low risk of resistance. On the other hand, there have been reports of treatment failure VL due to increasing resistance of Miltefosine in India. However, we do not have adequate diagnostic modalities to confirm resistance of anti-leishmania drugs. Since our patient’s response was excellent, it might be concluded that intermittent usage of these drugs might not develop resistance. However, this should be explored by robust studies to confirm the hypothesis.
